# Knowledge and beliefs of endocrine disruptors in pediatrics: all hands on deck!

**DOI:** 10.3389/fpubh.2024.1409215

**Published:** 2024-06-20

**Authors:** Aurélie Portefaix, Thomas Loppinet, Laura Tourvieilhe, Giuseppe Balice, Nathan de Veron de La Combe, Behrouz Kassai, Justine Bacchetta

**Affiliations:** ^1^Clinical Investigation Center P-1407, Department of General Pediatrics, Hospices Civils de Lyon, Lyon, France; ^2^Université Claude Bernard Lyon, UMR 5558, LBBE - EMET, CNRS, Lyon, France; ^3^Pôle de Psychologie Sociale (PôPS) - UMR1296 (Radiations, Défense, Santé, Environnement), INSERM, Université Lyon, Bron, France; ^4^Pediatric Nephrology Unit, INSERM1033 Research Unit and Lyon Est Medical School, Hospices Civils de Lyon, Lyon, France

**Keywords:** ED, pediatric health professionals, endocrine disruptor (ECD), pediatric health, knowledge beliefs

## Abstract

Endocrine disruptors (ED) are ubiquitous pollutants, possibly implicated in chronic disease. Exposure of vulnerable populations; including neonates, infants and children; must therefore be limited. Informing parents is now a public health challenge. We conducted a quantitative cross-sectional study at the Lyon Mother and child Hospital. We used questionnaires to assess the beliefs and knowledge about ED of parents and pediatric healthcare professionals in the pediatric ward in Lyon, France. A total of 746 questionnaires were completed: 444 for professionals and 302 for parents. The majority of both populations had already heard of ED but only 10% of parents and 5% of professionals felt sufficiently informed. Professionals answered better than parents (73% vs. 60%). The main source of information was similar: media. Only 20% of professionals had read a scientific article about ED and 4% have followed a training. Environmental exposure and EDs is an increasing concern for parents but specific knowledge remains scare for parents and professionals. Specific training is needed.

## 1 Introduction

The impact of the environment on population health is a growing concern for public policy and health professionals. According to the World Health Organization (1), approximately 24% of global mortality is due to the environment. Endocrine disruptors (ED) contribute to this mortality as chemical compounds ubiquitously present in the environment. There are suspected to contribute to the development of chronic diseases such as diabetes, obesity, precocious puberty, and fertility (2). Their mechanisms of action are not fully understood, but may include mimetic hormone effects, antagonistic effects, and epigenetic effects (3). However, these mechanisms can be different between EDs or have a cumulative effect. Taken together, these uncertainties and complexity limit studies of high-level of evidence. Recommendations are to limit and decrease exposures as much as possible.

Environmental safety is one parents’ primary concern, inducing many questions to Pediatric Health Professionals (PHP) in general, notably pediatricians, nurses, childcare assistants, and secretaries. Pregnancy and childhood are vulnerability windows, particularly in neonates, infants, and during puberty (4). Exposures during these periods are more at risk of engendering (long-term) health effects. Still, environmental exposure is a relatively recent concern: in France, dedicated teaching courses in medical schools are just emerging.

As PHPs, we should help parents to adopt good health behavior to preserve their children health, but previous studies have reported a lack of knowledge on these topics among health professionals, including midwives, obstetricians, general practitioners and health care professionals (5–8). However, very little data are available specifically for PHPs (9, 10), and parents (11, 12). Dedicated ED questionnaires are scarce; specific knowledge or representation are less evaluated (13).

Knowledge is not the only determinant of health behavior. Indeed, even though we all know that smoking kills people, some people keep smoking. Thus, an evaluation of knowledge and beliefs on ED is necessary to know from where we come and to improve both information and message delivery to the family. Our objectives were first to describe the knowledge and beliefs of both PHPs and parents, as well as their sources of information, using the same questionnaire, and second to search for factors of “better” answers.

## 2 Methods

We performed the STENDAL study, a cross-sectional quantitative study of parents and professionals at the Lyon Mother and Child Hospital, a tertiary pediatric university hospital (58,000 out-patient clinics, 68,000 hospitalizations and 85,000 visits at the pediatric emergency room yearly).

We proposed the study to all parents who referred their children at the out-patient clinics (medical and chirurgical). A student was present in the waiting rooms during the study period, to directly give parents information and hand-delivered questionnaire after agreement to participate. Parents filled the questionnaire directly. All professionals from the PHP, including physicians, nurses, assistant nurses, secretaries, administrative and research staff received the questionnaire through an individual email sent to their professional mailbox. They had 2 months to respond, and one reminder was sent. A total of 408 questionnaires were given to parents between June 30, 2022 and October 24, 2022. All 1,580 PHP of the Lyon Mother and Child Hospital received a personal email the 3rd of May 2023, and one personal remember the 15th of May 2023.

We constructed a 15-item standardized self-administered questionnaire (Supplementary Tables S1, S2) with one multiple-choice question and 14 true/false questions. The questionnaire was the same for professionals and parents. The questions concerned theoretical knowledge (questions 0, 1, 6, 10, 11, and 14) and beliefs (questions 2, 3, 4, 5, 7, 8, 9, 12, and 13) regarding EDs. The score was calculated from 15 questions. A point was awarded for correct answers, and 0 for incorrect or missing responses. Six questions evaluated the belief dimension, seven questions evaluated knowledge, one evaluated knowledge and belief and one question two different aspects of belief. Questions 2, 9, and 14 evaluated the confidence in the “natural” products. Questionnaires were anonymous and simple demographic data (i.e., profession, reasons for consultation, age of professional, rural or urban residency) were collected at the beginning of the questionnaire survey.

The study was approved by the Ethics Committee of Hospices Civils de Lyon (n° 2022022). Written information with correct answers were given to parents after receiving the questionnaire. An hospital meeting was organized to present the results of the survey to PHPs in October 2023.

Descriptive statistics were used to describe the responses. Qualitative data are reported as percentage and quantitative data as median. Correct responses rates were compared using nonparametric Kruskal-Wallis and Mann Whitney U tests. *p*-values presented are not adjusted for multiple testing and are not inferential. Analysis was conducted with R statistical software version 4.2.

## 3 Results and discussion

### 3.1 Results

In total, 746 questionnaires were completed. The response rate was 28% (*n* = 444) for PHP and 74% (*n* = 302) for parents.

**Figure 1:**
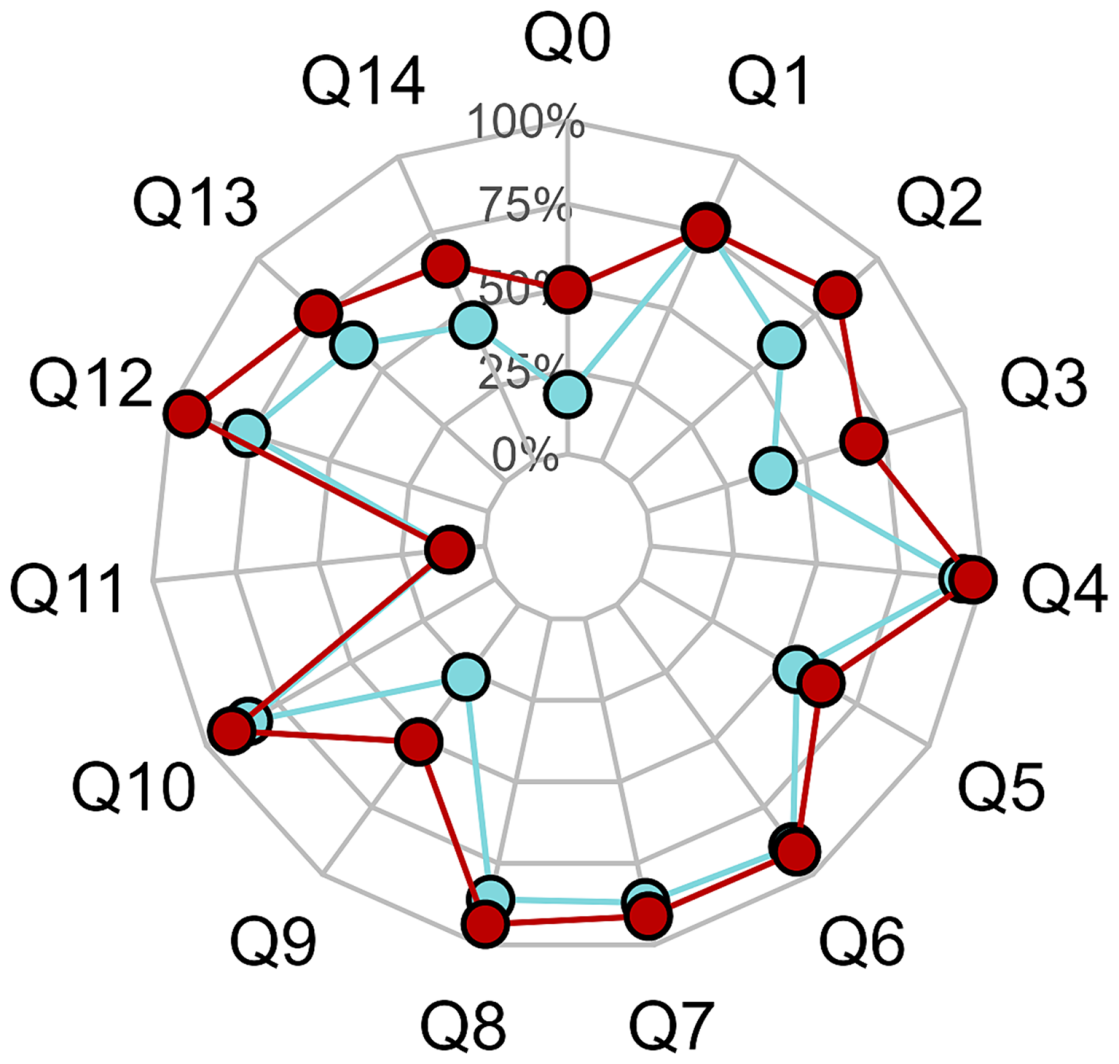
Percentage of correct answers of parents (blue points) and pediatric health professional (red).

**Figure 2:**
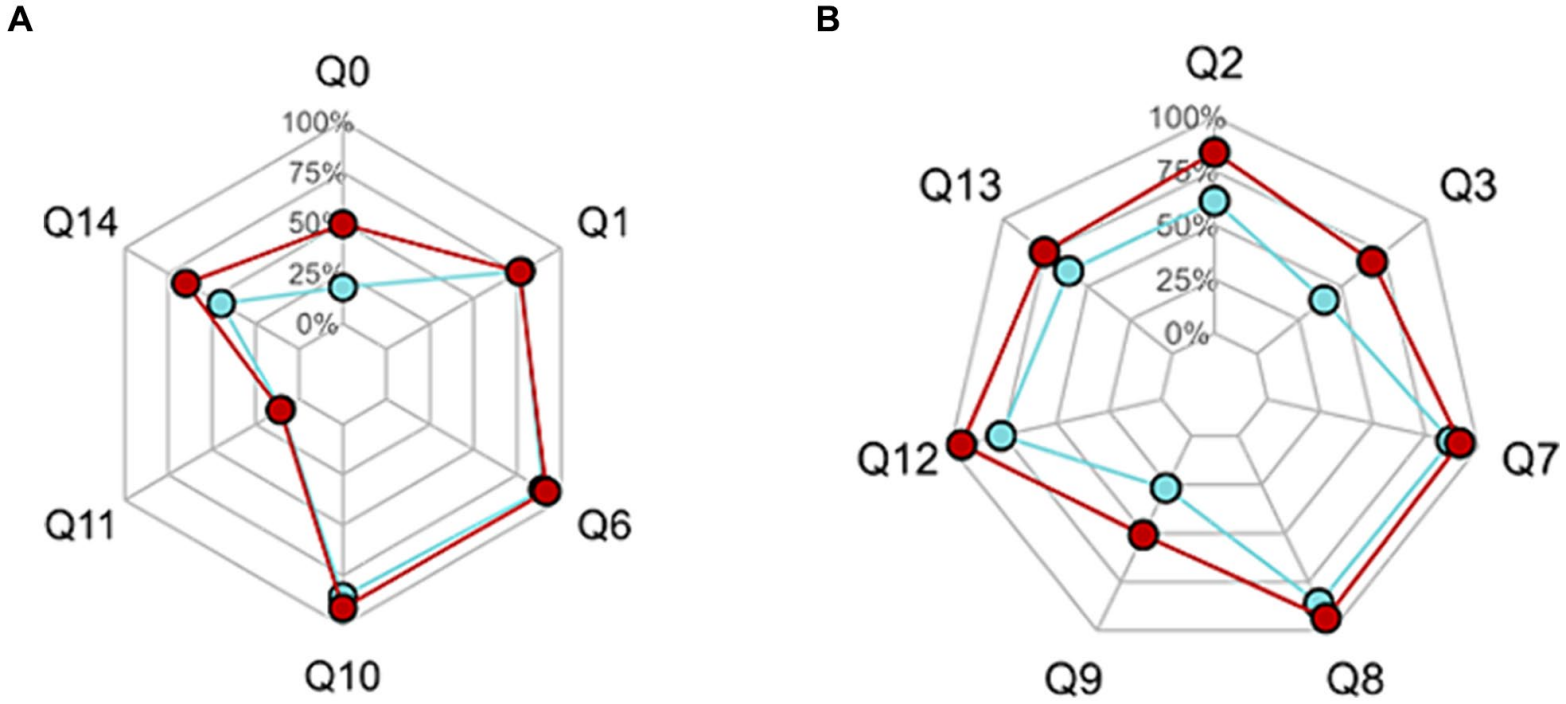
**(A)** Percentage of correct answers responses in knowledge questions. **(B)** Percentage of “good” responses in beliefs questions. Red points represent pediatric health professionals’ results. Blue points represent parents’ results.

The overall percentage of correct answers was 68%. It was significantly better in the PHPs’ group compared to the parents’ group (73% vs. 60%, respectively, *p* < 0.001). Proportions of correct answers to “knowledge” and “beliefs” questions were, respectively, 50 and 57% for parents, and 67 and 85% for PHPs. Rate of “good responses” to “belief” questions were 100% for medical doctor and 71.4% for the other professionals. The global (Figure 1) and specific responses (Figures 2A,B) of the two groups are displayed in the radar chart.

Questions 2, 9, and 14 related to confident in natural products have, respectively, 61, 27, and 45% of good answers in parents’ group and 84, 51, and 65% of good answers in PHPs’ group.

The highest proportion of correct answers was for the question on cosmetic products (question 4): 94% in the parents’ group and 97% in the PHPs’ group (*p* = NS). The lowest proportion of good answers was for the question on EDs mechanisms of action (question 11): 10% for parents and 11% for professionals (*p* = NS).

**Figure 3:**
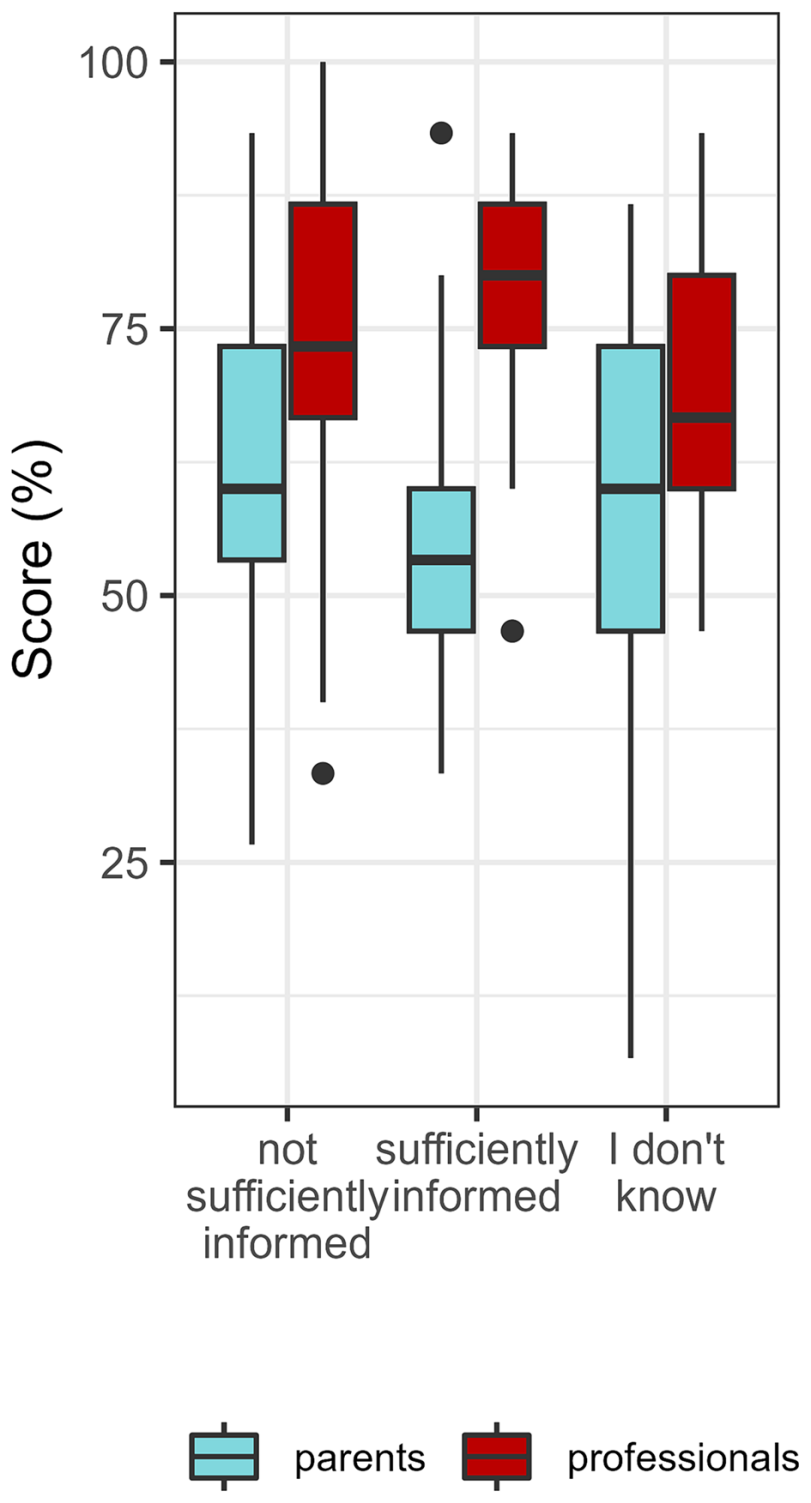
Correct answers from parents and professionals correct according to their perceived level of information about ED.

Overall, 66% of parents and 95% of PHPs had already heard of ED. This 66% of parents had a significantly better score than the others (67% vs. 53%, *p* < 0.001); similarly, this 95% of PHPs had a significantly better score (71% vs. 57%, *p* = 0.002). However, only 10% of parents and 5% of PHPs reported that they felt sufficiently informed, but they did not have a significantly better score than participants who felt insufficiently informed (Figure 3).

We have observed that 20% of both parents and PHPs systematically screened the product composition, while 15% never screened it. However, this screening was not linked to the proportion of correct answers. By univariate analysis, factors associated with a higher proportion of correct answers for parents were rural location and higher socio-professional categories. For PHPs, these factors were age under 35 years and being a physician.

**Figure 4:**
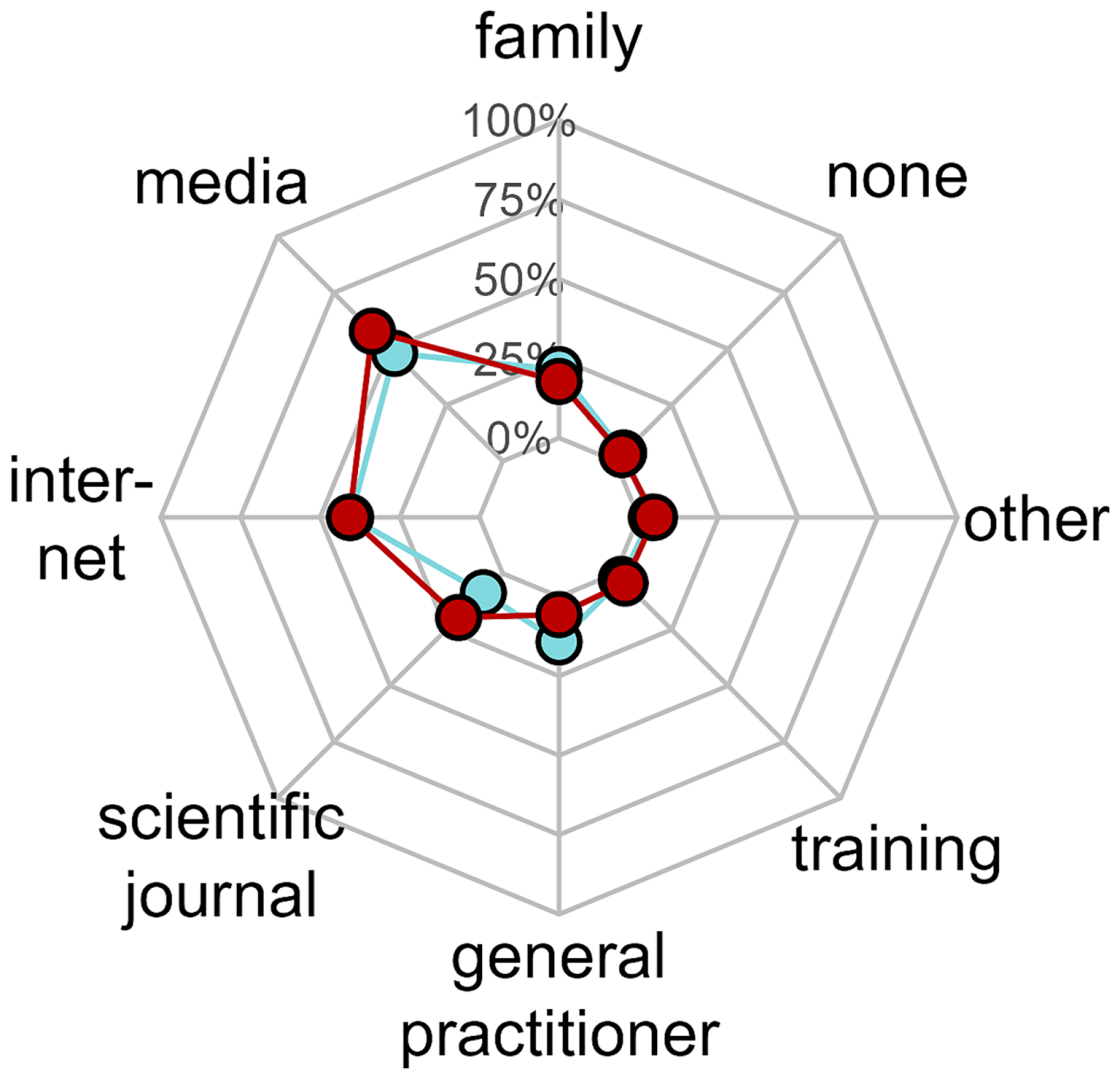
Information or training resources about EDs. Red points represent pediatric health professionals, blue points represent parents.

Information sources were similar between parents and PHPs, the most frequent one being media (television or radio) and the web for 70% of parents and 80% of PHPs; PHPs used mostly “general public” resources. Only 20% of PHPs read scientific papers, and 4% have followed a training on EDs (Figure 4).

### 3.2 Discussion

Knowledge on EDs is not optimal both for parents and PHPs. The main strengths of this study were a significant number of answers and an evaluation of both parents and PHPs with the same questionnaire. All professionals interacting with parents and children were represented, thus reinforcing the strength of the study. However, PHPs’ knowledge and beliefs were not optimal, and most of them were not trained on EDs.

Our rates of responses are good, close, or better than other reports in the literature, i.e., 11–30% (5, 6, 9). Reasons for obtaining a satisfactory response rate are probably multiple and intricated: the increasing concern about environmental exposures, the availability of researchers for parents with a hand-delivered questionnaire, and the individual mail for the PHPs in a pediatric hospital in which the principal investigator of the study is the senior physician responsible for the pediatric research centre.

Some factors were linked to the profile of “good responders.” For parents these were rural locations and higher socio-professional categories. In rural areas, parents and their children are exposed to specific pollutants such as pesticides and information on this type of environmental exposures is probably more spread. Higher socio-professional categories often have better level of education and better access to information. Furthermore, for the lower socio-professional categories, environmental exposures are not a priority in daily life, even though they are the population most exposed. For professionals, the profile of “good responders” was under 35 years of age and being a doctor. Young professionals are more sensitive to environmental exposures and climate change. Good responses from doctor are linked to better answers to beliefs questions (100% for medical doctor vs. 71.4% for other professionals). Even though they have not received a specific training on EDs, the training provided during their medical training has enabled doctors to acquire a critical mind, which may explain their better responses to beliefs questions.

This single-center study has nevertheless several limitations, by design. As a result, only professionals and parents from patients followed in a tertiary university hospital participated. One may assume that liberal practitioners are more concerned and informed because they receive more questions from parents, with less severely ill children. Also, responders probably felt more concerned with the questions of environmental exposure than parents and PHPs who did not participate. Therefore, we could expect that responses in general populations will be even worse. In addition, we used a non-validated personal questionnaire.

In our study, only 10% of parents and 5% of PHPs considered they are sufficiently informed about EDs. This result is congruent with a previous study reporting that only 11% of perinatal health professionals were sufficiently trained and informed (14). Another French study also found 82% of insufficiently informed professionals (5). The lack of specific training for health professionals on the topic has also been reported in many studies worldwide (10, 14).

The worst answers were about “natural” products. In fact, each question with the term “natural” automatically generated an analysis of “healthy” in parents’ mind but also, however in a lesser extent, in PHPs. As described in human health science publications, instructional messages are not an optimal way to inform both parents and PHPs (15). Dichotomist ideas such as “natural is good” and “industrial is bad” have to be avoided; beliefs must be explored in order to give better messages to the population. Moreover, it may be relevant to analyze in future studies in which state of mind PHPs who are also parents position themselves.

More worrying is the way used by professionals to get information, as already descripted in 2001 (9). There is an urgent need for professionals to be better informed. To achieve this, specific training courses and recommendations from medical scientific societies are required. For example, the International Federation of Gynecology and Obstetric guidelines refer to the presence of heavy metal and EDs in prenatal vitamins and recommends reducing the exposure. These guidelines are easily accessible for the professionals. Conversely, information in the media is often perceived as stressful and incomprehensible by parents (8).

In conclusion, we show that, despite the fact that the risk of environmental exposure and EDs is an increasing concern for parents, the specific knowledge remains scarce both for parents and PHPs. PHPs need to be trained on the topic, so as to provide optimal advice to families.

## Data availability statement

The raw data supporting the conclusions of this article will be made available by the authors, without undue reservation.

## Ethics statement

The studies involving humans were approved by the Ethics Committee of Hospices Civils de Lyon n° 2022022. The studies were conducted in accordance with the local legislation and institutional requirements. The participants provided their written informed consent to participate in this study.

## Author contributions

AP: Conceptualization, Methodology, Project administration, Writing – original draft. TL: Data curation, Formal analysis, Writing – review & editing. LT: Investigation, Project administration, Writing – review & editing. GB: Investigation, Software, Writing – review & editing. NV: Investigation, Software, Writing – review & editing. BK: Supervision, Writing – review & editing. JB: Supervision, Writing – review & editing.
