# Supplementary material for: Knowledge and beliefs of endocrine disruptors in pediatrics: all hands on deck!
Source: Front Public Health. 2024 Jun 20;12:1409215. doi: 10.3389/fpubh.2024.1409215 (PMC11225406; doi:10.3389/fpubh.2024.1409215)
Supplement: Supplementary file 1 [file Table_1.DOCX]

Supplementary Material

Table 1: parents’ survey

Parents’ survey – Stendal study

The aim of this questionnaire is to assess your knowledge of endocrine disruptors as parents. There are no right or wrong answers. Your answers will be collected anonymously. You have the right to refuse to take part.

| **Demographic data** |
| --- |
| You live: (only one answer required) ☐ in the country ☐ in the city  Your professional category:  ☐ Farmer ☐ Employee  ☐ Craftsmen, shopkeepers, company managers ☐ Manual workers  ☐ Managers, higher intellectual professions ☐ Intermediate professions  ☐ Other : ________________________________ ☐ Unemployed |
| **About your consultation today** |
| Have you completed this questionnaire before? ☐ This is the first time  ☐ This is the second time  Age of child coming for consultation: __ __ months OR __ __ years  Specialist consulted :  ☐ Nephrologist ☐ Rheumatologist ☐ Neurologist ☐ Endocrinologist ☐ Gastroenterologist  ☐ Haematologist ☐ Pneumologist ☐ Allergist ☐ Other, specify : ____________________________ |

| **Your information** | | | |
| --- | --- | --- | --- |
| Have you ever heard of endocrine disruptors? | □ Yes  □ No | | |
| What is your main source of information? (only one answer expected) | □ Family or friends  □ Mainstream media (TV, press, radio)  □ Internet  □ Scientific journals  □ Our medical doctor  □ Other : ___________________________  □ None | | |
| Do you think you are sufficiently informed? | □ Yes  □ No  □ I don’t know | | |
| Do you look at the composition of products before you buy them? | □ Yes, systematically  □ Yes, from time to time  □ Yes, rarely  □ No, never  □ Not concerned | | |
| **Our knowledge** | | | |
| You may come into contact with endocrine disruptors through : (one or more answers expected)  □ Contact with people who are often exposed to endocrine disruptors  □ Food containers □ Pesticides  □ Medicines □ None  □ Hygiene and cosmetic products □ I don't know | | | |
|  | | | |
| **TRUE/FALSE** | | | |
| 1. Baby food is regulated with regard to its composition of potential endocrine disruptors. | | **□ True** | **□ False** |
| 2. Lavender essential oil and tea tree essential oil are natural products and therefore pose no risk. | | **□ True** | **□ False** |
| 3. The aluminium contained in vaccines is dangerous. | | **□ True** | **□ False** |
| 4. The use of cosmetic products (make-up, nail varnish, hair dye) by children should be limited as much as possible, as they contain additives that may be endocrine disruptors. | | **□ True** | **□ False** |
| 5. Exposure to endocrine disruptors can lead to early puberty (before the age of 9 for girls and 10 for boys), but this has no consequences for children. | | **□ True** | **□ False** |
| 6. When painting inside my home, I have to air the room for several days before using it. | | **□ True** | **□ False** |
| 7. Simple measures (airing out for at least 15 minutes a day, avoiding reheating food in plastic containers, favouring seasonal produce, etc.) can help limit exposure to endocrine disruptors. | | **□ True** | **□ False** |
| 8. For household products, simple products such as white vinegar, Marseille soap and bicarbonate of soda can reduce exposure to endocrine disruptors. | | **□ True** | **□ False** |
| 9. Home-made products are always "safer" than shop-bought products. | | **□ True** | **□ False** |
| 10. Pregnant women and newborns are particularly vulnerable to endocrine disruptors. | | **□ True** | **□ False** |
| 11. The more endocrine disruptors we expose ourselves to, the more harmful the consequences. | | **□ True** | **□ False** |
| 12. Over-the-counter products sold in pharmacies and drugstores are always safe. | | **□ True** | **□ False** |
| 13. The health effects of endocrine disruptors are easy to prove and well-documented. | | **□ True** | **□ False** |
| 14. Endocrine disruptors are synthetic products, not natural compounds. | | **□ True** | **□ False** |

Table 2: survey of paediatric health professionals

Survey of paediatrics health professionals – STENDAL study

*The aim of this questionnaire is to assess your general knowledge of endocrine disruptors as paediatric healthcare professionals. Your answers will be collected anonymously. You have the right to refuse to take part. At the end of the questionnaire, you will be given an information leaflet.*

| **General Informations** |
| --- |
| You live: (only one answer required) ☐ in the country ☐ in the city  Your age range: ☐ 20-29 years ☐ 30-45 years ☐>45 years  Your main mode of transport to work is: ☐ car ☐ public transport ☐ bicycle/scooter ☐ walking  You are:  ☐ Nursery assistant ☐ Care assistant  ☐ Nurse ☐ Nursery nurse  ☐ Dietician ☐ Physiotherapist  ☐ Senior doctor ☐ Resident  ☐ Care executive  ☐ Liberal paediatrician  ☐Other : ________________________________  You are working: :  ☐ Hospitalisation ☐ Medicine  ☐ Day hospitalisation ☐ Surgery  ☐ Consultation ☐ Private Practice  ☐ Other, specify  : ___________________________ |
|  |

| ***Your Information*** | | | |
| --- | --- | --- | --- |
| Have you ever heard of endocrine disruptors? | □ Yes  □ No | | |
| What is your main source of information? (only one answer expected) | □ Family or friends  □ Mainstream media (TV, press, radio)  □ Internet  □ Scientific journals  □ Our medical doctor  □ Other : ___________________________  □ None | | |
| Do you think you are sufficiently informed? | □ Yes  □ No  □ I don’t know | | |
| Do you look at the composition of products before you buy them? | □ Yes, systematically  □ Yes, from time to time  □ Yes, rarely  □ No, never  □ Not concerned | | |
| **Our knowledge** | | | |
| You may come into contact with endocrine disruptors through : (one or more answers expected)  □ Contact with people who are often exposed to endocrine disruptors  □ Food containers □ Pesticides  □ Medicines □ None  □ Hygiene and cosmetic products □ I don't know | | | |
|  | | | |
| **TRUE/FALSE** | | | |
| 1. Baby food is regulated with regard to its composition of potential endocrine disruptors. | | **□ True** | **□ False** |
| 2. Lavender essential oil and tea tree essential oil are natural products and therefore pose no risk. | | **□ True** | **□ False** |
| 3. The aluminium contained in vaccines is dangerous. | | **□ True** | **□ False** |
| 4. The use of cosmetic products (make-up, nail varnish, hair dye) by children should be limited as much as possible, as they contain additives that may be endocrine disruptors. | | **□ True** | **□ False** |
| 5. Exposure to endocrine disruptors can lead to early puberty (before the age of 9 for girls and 10 for boys), but this has no consequences for children. | | **□ True** | **□ False** |
| 6. When painting inside my home, I have to air the room for several days before using it. | | **□ True** | **□ False** |
| 7. Simple measures (airing out for at least 15 minutes a day, avoiding reheating food in plastic containers, favouring seasonal produce, etc.) can help limit exposure to endocrine disruptors. | | **□ True** | **□ False** |
| 8. For household products, simple products such as white vinegar, Marseille soap and bicarbonate of soda can reduce exposure to endocrine disruptors. | | **□ True** | **□ False** |
| 9. Home-made products are always "safer" than shop-bought products. | | **□ True** | **□ False** |
| 10. Pregnant women and newborns are particularly vulnerable to endocrine disruptors. | | **□ True** | **□ False** |
| 11. The more endocrine disruptors we expose ourselves to, the more harmful the consequences. | | **□ True** | **□ False** |
| 12. Over-the-counter products sold in pharmacies and drugstores are always safe. | | **□ True** | **□ False** |
| 13. The health effects of endocrine disruptors are easy to prove and well-documented. | | **□ True** | **□ False** |
| 14. Endocrine disruptors are synthetic products, not natural compounds. | | **□ True** | **□ False** |
